# Does the surgical sequence of lumbar spine fusion and total hip arthroplasty affect the dislocation and revision risk? A meta‑analysis and systematic review

**DOI:** 10.1186/s10195-026-00943-5

**Published:** 2026-06-12

**Authors:** Fuze Liu, Chengqi Jia, Xiangdong Wu, Yixin Zhou

**Affiliations:** 1 Department of Orthopaedic Surgery, Beijing Jishuitan Hospital, Capital Medical University, National Center for Orthopaedics, Beijing, China; 2 Beijing Research Institute of Traumatology and Orthopaedics, Beijing Jishuitan Hospital, Capital Medical University, Fourth Clinical College of Peking University, National Center for Orthopaedics, Beijing, China

**Keywords:** Total hip arthroplasty, Lumbar spine fusion, Dislocation, Revision

## Abstract

**Background:**

Patients who underwent primary total hip arthroplasty (THA) after lumbar spine fusion (LSF) had a higher incidence of complications compared with those who had not previously undergone LSF. This study aimed to determine whether the timing of THA before or after LSF impacts the incidence of THA complications.

**Methods:**

Following the Preferred Reporting Items for Systematic reviews and Meta-Analyses (PRISMA) guidelines, we searched PubMed, Web of science, Embase, and Cochrane Library from their inception until 1 October 2025. Data from all eligible published studies meeting the inclusion criteria were extracted and subsequently analyzed using a random-effects model to calculate the rates of all-cause revision, dislocation, periprosthetic joint infections, and aseptic loosening.

**Results:**

Our analysis included 10 studies involving 27,605 patients who underwent THA followed by LSF and 32,002 patients who received LSF prior to THA. There are no significant differences in the rates of dislocation (odds ratio [OR] 1.19, 95% confidence interval (CI) 0.73–1.86, *p* = 0.45), periprosthetic joint infections (OR 0.91, 95% CI 0.52–1.62, *p* = 0.76), and aseptic loosening (OR 0.89, 95% CI 0.74–1.08, *p* = 0.23) between the two surgical sequences, while for all-cause revision, the OR was (OR 1.01, 95% CI 0.70–1.47, *p* = 0.94), which reached statistical significance.

**Conclusions:**

Compared with the patients undergoing THA after LSF, no significant difference for complication rates was observed in patients with LSF after THA.

**Supplementary Information:**

The online version contains supplementary material available at 10.1186/s10195-026-00943-5.

## Introduction

Hip-spine syndrome, characterized by the co-existence of degenerative pathologies in the hip and lumbar spine, often presents with overlapping symptoms, complicating the diagnosis and treatment [1, 2]. Total hip arthroplasty (THA) and lumbar spinal fusion (LSF) are effective interventions for degenerative disease, offering significant pain relief and functional improvement [3, 4]. However, when patients have both hip and spine syndromes, the recommended sequence of THA and LSF, which decreases the impact on complications of THA, is still unclear.

During the transition from standing to sitting, a spine with normal flexibility facilitates pelvic retroversion, thereby increasing the functional anteversion of the acetabulum [5–7]. This mechanism ensures sufficient femoral head coverage within the socket as the hip flexes. By contrast, lumbar fusion induces spinal stiffness, further restricting the mobility of the pelvis, which potentially elevates the risk of impingement and impacts the stability of THA [8–10]. While Parilla reported comparable dislocation rates irrespective of whether THA was performed before or after LSF, a study by Malkani et al. demonstrated that patients with LSF prior to THA faced a higher risk of dislocation relative to those in whom LSF was performed at least 5 years after THA [11, 12]. A meta-analysis reported that there was no difference between the two sequences in the complications [13]. However, only four studies finally included the analysis, and three out of them relied on institutional databases, which have an underreporting of complications [13]. As a result, the optimal surgical sequence for treating patients with concurrent hip and spine pathology to minimize such risks remains uncertain. This study was therefore designed to investigate whether the timing of LSF and THA influences the incidence of THA complications.

## Methods

This meta-analysis was performed in accordance with the Preferred Reporting Items for Systematic Reviews and Meta-Analyses Statement (PROSPERO number: CRD420251239562), adhering to the Assessing the Methodological Quality of Systematic Reviews guidelines [14, 15].

### Search strategy and identification of studies

A systematic search of electronic databases (PubMed, Web of Science, Embase, Cochrane Library) was performed from database inception to October 1st 2025, with specific keywords “Total hip arthroplasty” “Lumbar spine fusion,” as shown in Supplement file 2. Eligibility assessment was performed independently by two reviewers, who examined the titles, abstracts, and relevant full-text articles.

### Inclusion and exclusion criteria

Included studies must meet the following criteria: (1) study population must be THA patients undergoing LSF either before or after the arthroplasty; (2) study must include the rates of THA complications; (3) for the cohort receiving LSF after THA, no THA complications occurred between the initial THA and the subsequent LSF. Studies were excluded if they (1) were abstracts, letters, reviews, or case reports; (2) had repeated data; (3) were nonEnglish publications; and (4) studies that did not provide a direct comparison of complications between the “THA-after-LSF” and “THA-before-LSF” groups.

### Data extraction and quality of studies

Data extraction was completed by two investigators independently, including study design, first authors, publication year, sample size, interval time between two surgeries, follow-up duration, the rates of all-cause revision, dislocations, periprosthetic joint infections (PJI), and aseptic loosening. The methodological quality of the included articles was appraised with the Methodological Index for Nonrandomized Studies (MINORS), also by two independent investigators. 12 criteria were used to evaluate the involving studies. A 3-point scoring system was applied to each criterion, defined as follows: 0 (not reported), 1 (inadequately reported), and 2 (adequately reported). The ideal total score for a study is 24. A consensus between both reviewers was required for all inclusion decisions.

### Statistical analysis

The primary effect measure for this meta-analysis was the odds ratio (OR) along with its 95% confidence intervals. To synthesize the data, both fixed-effects and random-effects models were employed for comparison. The fixed-effects model operates under the assumption that the true treatment effect is uniform across all included studies, whereas the random-effects model allows for and accounts for potential inter-study variation. Heterogeneity across the included studies was quantified using the *I*^2^ statistic, with values of 25, 50, and 75% conventionally representing low, moderate, and high heterogeneity, respectively [16]. Given the anticipated clinical and methodological diversity among the included studies, a random-effects model was applied to present the results. A leave-one-out sensitivity analysis was performed to evaluate the robustness of the pooled estimate for the rates of all-cause revision, dislocation, PJI, and aseptic loosening. Furthermore, subgroup analyses were conducted to explore the influence of interval time and study design. Because of the deficiency of raw data, the potential confounding factors, such as the number of fusion segments and follow-up times, cannot be further analyzed. And a *p*-value ≤ 0.05 is considered statistically significant. The statistical analyses were conducted using the Rev Man 5.4 program.

## Results

### Literature search

**Fig. 1:**
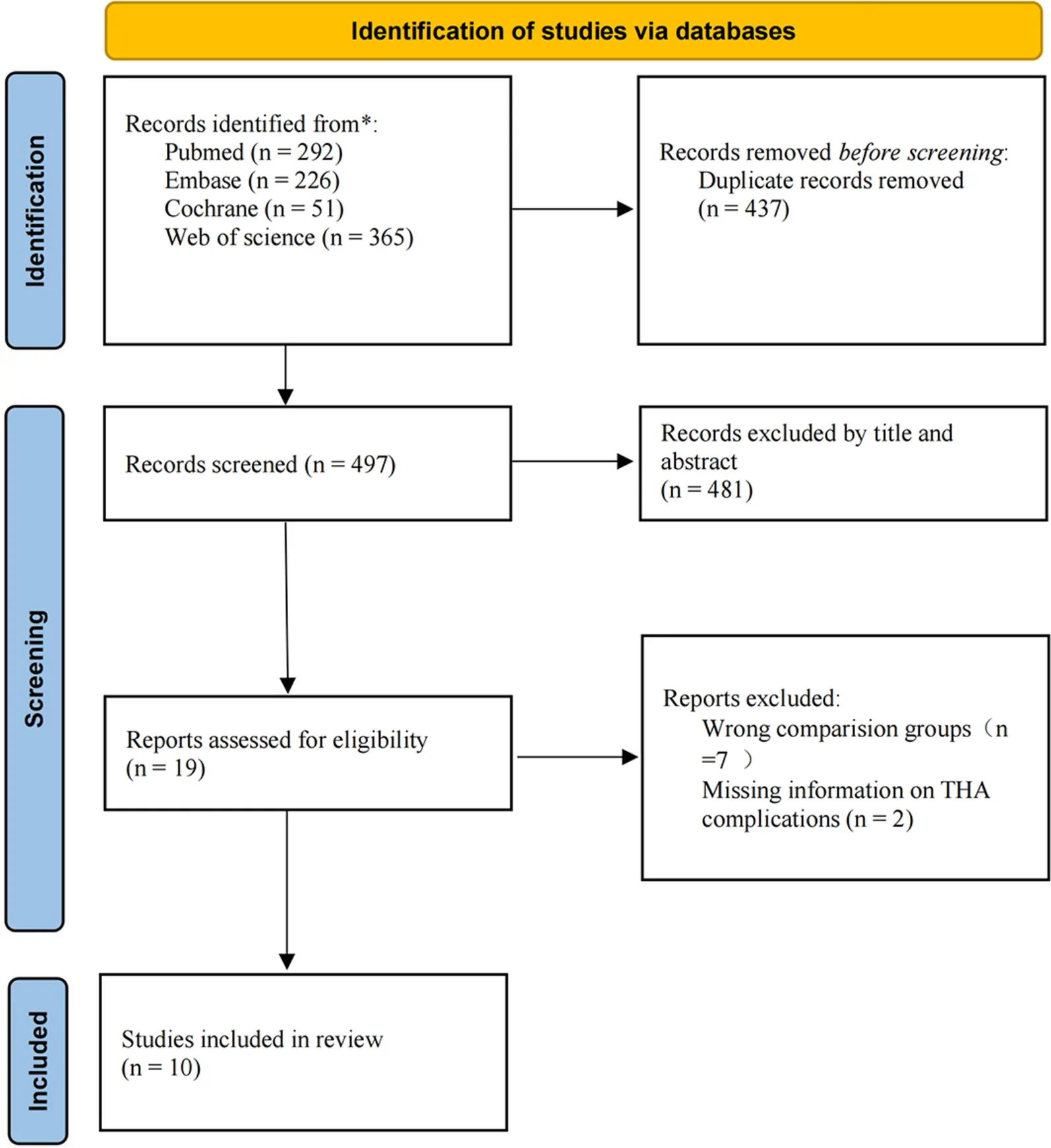
Flow chart summarizing results of the literature search

The process of literature selection is visually demonstrated in Fig. 1. A total of 934 studies were identified in the database search. After removal of 437 duplicates, the titles and abstracts of the remaining 497 studies were screened, leading to the exclusion of 481 irrelevant reports. Subsequently, a full-text review of the 19 potentially eligible articles was conducted, and 10 studies met all criteria for final inclusion.

### Assessment of quality

The MINORS score of the involved studies ranged from 16 to 20, as shown in Supplement file 1.

### Clinical characteristics

A total of 27,605 prior THA and 32,002 subsequent THA were identified. For the ten studies included, six studies utilized database information, while the other four studies relied on single-institution retrospective clinical records. The interval time between two surgical procedures varied between studies, and the fusion segments have only been documented in three studies. Comprehensive details are presented in Table 1.

**Table 1:** Clinical characteristics of included studies

Article	Year	Data source	Area	Number of patients		Interval time between procedures (y)		Number of spinal levels fused		All-cause revision		Dislocation		Periprosthetic joint infection		Aseptic loosening	
				1-THA	1-LSF	1-THA	1-LSF	1-THA	1-LSF	1-THA	1-LSF	1-THA	1-LSF	1-THA	1-LSF	1-THA	1-LSF
Bala	2019	Database	America	10,482	13,102					384	745	174	608	174	352	200	220
Grammatopoulos	2019	Single center	England	26	21	4.1	2.8	1.4	1.9	2	2	1	0	2	2	1	1
Parilla	2019	Single center	America	62	73	2.2	2.6					4	7			2	3
Yang	2020	Database	America	1356	2016	1.7	1.4			121	152	73	66	89	63		
Eneqvist	2024	Database	Sweden	4016	3892					130	87						
Andah	2021	Single center	America	67	42			2.43	2.97			8	0				
Welling	2023	Database	America	661	1429	< 1	< 1					30	51				
Fan	2025	Single center	China	85	98	5.48	5.52	2.65	2.27			3	2	5	2	5	7
Mohamed	2023	Database	America	4510	4510					203	220	180	262				
Zhang	2023	Database	America	6340	6819	< 2	< 2					161	314	348	343		

### Complications

**Fig. 2:**
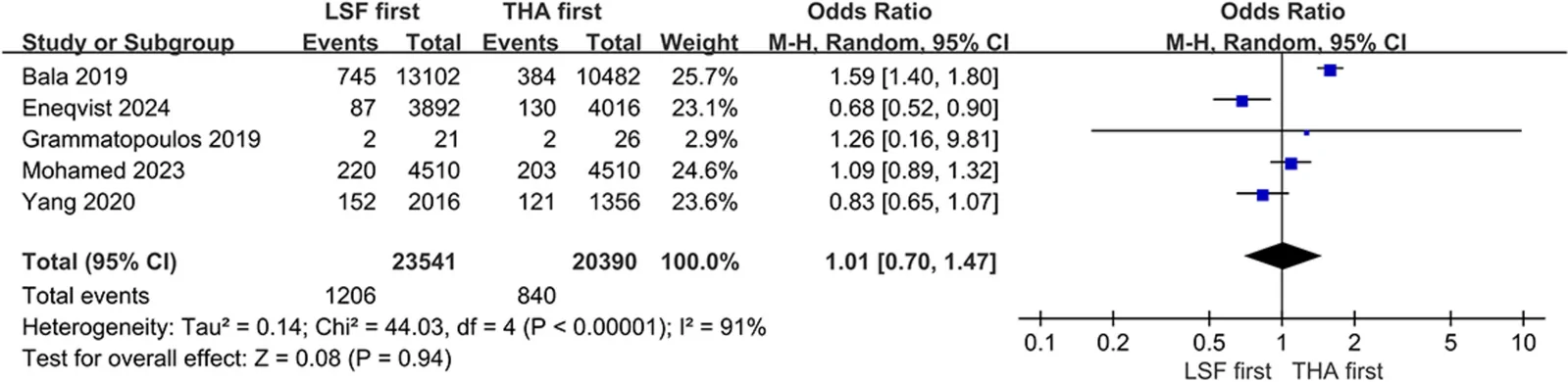
All cause revisions

**Fig. 3:**
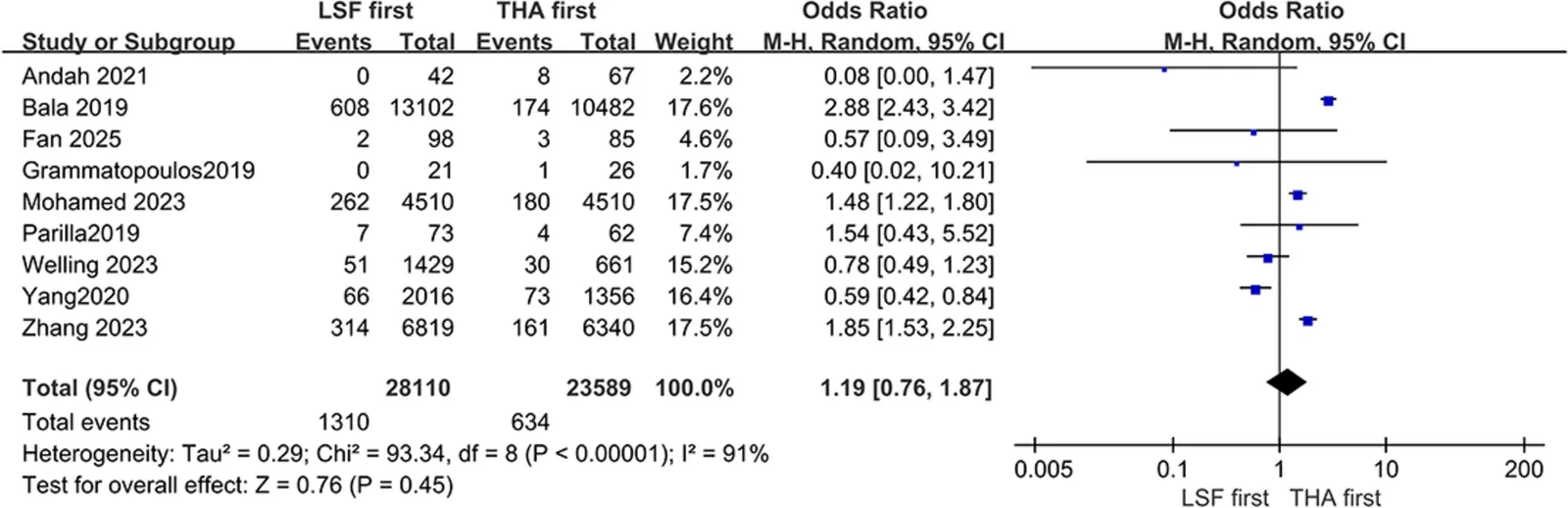
Dislocation

**Fig. 4:**
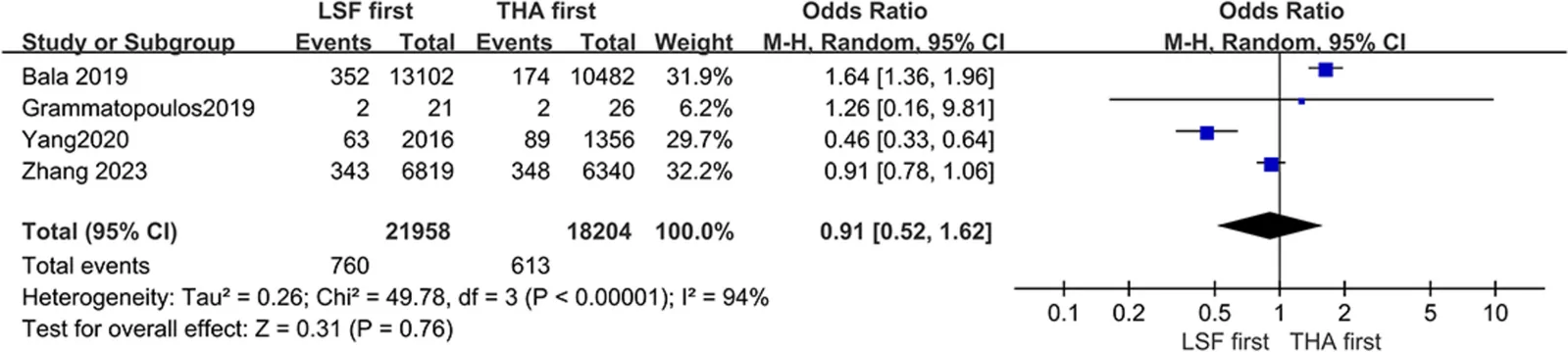
Peri-prosthetic joint infections

**Fig. 5:**
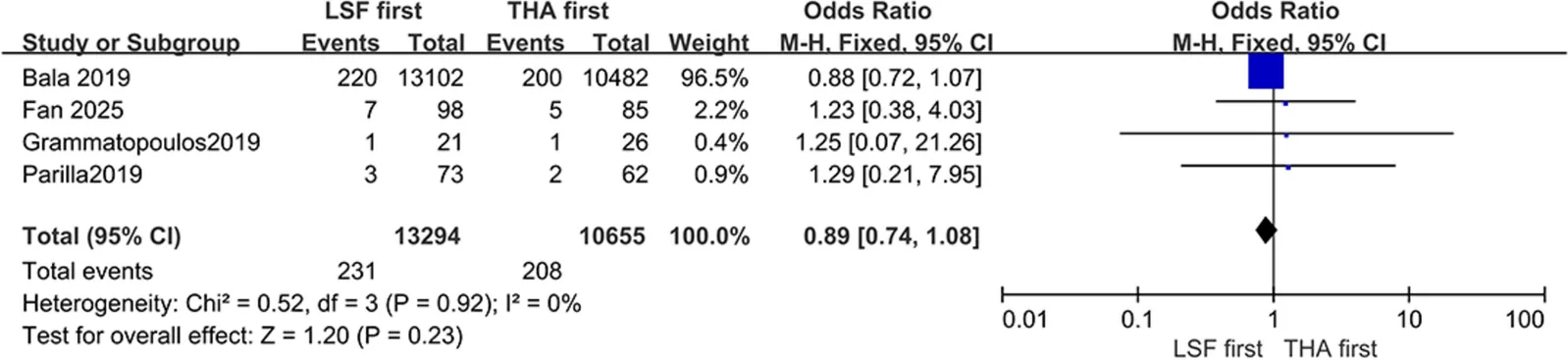
Aseptic loosening

No statistically significant differences were observed between the cohorts for the outcomes of revision (odds ratio [OR] 1.01, 95% CI 0.70–1.47, *p* = 0.94), PJI (OR, 0.91, 95% CI 0.52–1.62, *p* = 0.76), dislocation (OR, 1.19, 95% CI 0.73–1.86, *p* = 0.45), or aseptic loosening (OR, 0.89, 95% CI 0.74–1.08, *p* = 0.23), as detailed in Figs. 2, 3, 4 and 5. This finding indicates that there is no difference in revision, PJI, dislocation, and aseptic loosening between patients who underwent LSF before THA and patients who received THA followed by LSF. The sensitivity analysis revealed that the results remained robust upon the sequential omission of any single study, as seen in Supplement file 3.

### Subgroup analysis

The results of the subgroup analyses are presented in Table 2. When considering the interval time between the two surgical procedures, the rate did not differ significantly for dislocation, with interval time < 2 years (OR, 0.96, 95% CI 0.43–2.15, *p* = 0.93) and > 2 years (OR 1.00, 95% CI 0.37–2.72, *p* = 0.99). Regarding PJI, an OR of 0.66 (95% CI 0.34–1.29, *p* = 0.22) was observed in the group with an interval time < 2 years, while an OR of 0.57 (95% CI 0.16–2.06, *p* = 0.39) was noted in the group with an interval time > 2 years. When stratified by data source, the analysis revealed the following results. For all-cause revision, the odds ratios were 0.98 (95% CI 0.55–1.72, *p* = 0.93) for database-derived studies and 1.26 (95% CI 0.16–9.81, *p* = 0.82) for single-center studies, respectively. Similarly, for dislocation, the OR was 1.33 (95% CI 0.82–2.17, *p* = 0.25) in databases and 0.65 (95% CI 0.20–2.10, *p* = 0.47) in single centers. For periprosthetic joint infection (PJI), the corresponding values were OR 0.89 (95% CI 0.49–1.62, *p* = 0.71) and OR 0.57 (95% CI 0.16–2.06, *p* = 0.39). Finally, for aseptic loosening, database studies showed an OR of 0.88 (95% CI 0.72–1.07, *p* = 0.19), while single-center studies showed an OR of 1.25 (95% CI 0.49–3.19, *p* = 0.64).

**Table 2:** Subgroup analysis of complications

Complications	Subgroup	Number of studies	1-LSF		1-THA		Odds ratio	95% CI	Heterogeneity *I*^2^(%)	*p*-Value
			Events	Total	Events	Total				
Interval time										
Dislocation	< 2 years	3	431	10,264	264	8357	0.96	0.43–2.15	95	0.93
	> 2 years	3	9	192	8	173	1.00	0.37–2.72	0	0.99
PJF	< 2 years	2	406	8835	437	7696	0.66	0.34–1.29	93	0.22
	> 2 years	2	4	119	7	111	0.57	0.16–2.06	0	0.39
Data source										
All-cause revision	Database	3	984	19,010	635	15,854	0.98	0.55–1.72	95	0.93
	Single center	1	2	21	2	26	1.26	0.16–9.81	Not applicable	0.82
Dislocation	Database	5	1301	27,876	618	23,349	1.33	0.82–2.17	95	0.25
	Single center	4	9	234	16	240	0.65	0.20–2.10	25	0.47
PJI	Database	3	758	21,937	611	18,178	0.89	0.49–1.62	96	0.71
	Single center	2	4	119	7	111	0.57	0.16–2.06	0	0.39
Aseptic loosening	Database	1	220	13,102	200	10,482	0.88	0.72–1.07	Not applicable	0.19
	Single center	3	11	192	8	173	1.25	0.49–3.19	0	0.64

## Discussion

Because of the coexistence of lumbar and hip degenerative diseases, it is common for patients to receive both THA and LSF. However, the optimal sequence of whether THA or LSF should be performed first to decrease the rates of THA complications remains controversial. Our study compared the complications of THA in the two surgical sequences. The results of the analysis demonstrated that the rates of revision dislocation, PJI, and aseptic loosening have no significant difference between the two groups. This suggests that there is no big difference between which LSF and THA perform first. Besides, subgroup analysis stratified by the interval between surgeries (< 2 years versus > 2 years) revealed comparable complication rates between the group of THA after LSF and LSF after THA. Meanwhile, considering the data source, we performed comparisons of THA complication rates in the database group and single-center group, and no statistically significant difference was observed.

It has been widely reported that patients with a history of LSF usually have worse functional outcomes and higher risks of THA complications, including dislocation, revision, and the consumption of opioids [17–19]. Besides, compared with patients who undergo THA, patients who do not undergo THA have a lower risk of LSF complications, such as adjacent segment disease [20]. During the standing-to-sitting position, the mass of the body is transferred through the hip. Patients with stiff spines or spine surgical fusion are characterized by reduced lumbar lordosis (LL) and excessive posterior pelvic tilt (PT), and the limitation of spinopelvic mobility needs more hip flexion to compensate [12, 21, 22]. However, the increased hip flexion contributes to a higher risk of femoroacetabular impingement and posterior dislocation of the femoral head [10, 23, 24]. Given the established link between LSF and THA outcomes, the optimal sequence of these procedures requires careful consideration in patients with hip-spine syndrome to minimize complications.

It is suggested that LSF after THA leads to a decreased dislocation rate in some studies, compared with THA after LSF [12, 20, 25–27]. For patients with LSF after THA, limited LL and a stiff spine are not enough to alter functional anteversion for a well-performed THA [7]. It has also been found that LSF after THA has positive effects on PT and sacral slope (SS), suggesting a better pelvic alignment [28]. The timing of the dislocation was also different in the two surgical sequences, as THA after LSF has a higher proportion of early dislocations, and LSF after THA usually has a late dislocation [7, 12]. The patients with THA first have enough time for bony ingrowth, soft-tissue healing, and muscle strengthening to adapt to the altered spinopelvic biomechanics [7]. Moreover, a better functional outcome and lower risk of pneumonia and PJI were observed in LSF after THA at follow-up [26, 29].

However, a clinical concern is that performing LSF after THA could further stiffen the spinopelvic unit, which may adversely affect the stability of the preexisting THA and increase dislocation rates [30, 31]. Higher rates of dislocation and revision were obtained in patients with LSF after THA, compared with the patients undergoing LSF before THA [30]. It has been demonstrated that concurrent spine pathology, such as degenerative disc disease, can decrease spine flexion and increase femoroacetabular flexion during the standing-to-sitting position, resulting in impingement and dislocation [10]. Besides, the increasing anteversion is another risk factor contributing to posterior impingement, and spinal correction is useful to reduce acetabular anteversion [30, 32]. As a result, performing LSF before THA to improve spinal alignment and the definitive acetabular component can be positioned in a manner that is inherently more stable under the patient’s new spinopelvic mechanics [13]. A key determination for surgeons is the postoperative time interval following LSF beyond which further compensatory changes in PT are minimal and predictable, ensuring long-term functional anteversion remains within a target range. Given that the alteration of the radiologic parameter, such as PT, which influences the functional anteversion, it is necessary to consider the postoperative time interval following LSF. As the alteration of PT primarily occurs predominantly in the first year after LSF, a 1-year waiting period before subsequent THA is justified [11]. Besides, a high and prolonged opioid utilization was observed in patients with THA before LSF [30]. And addressing lower back pain with LSF firstly may result in decreased utilization of opioids [30].

Patients who experienced dislocations usually have worse spinopelvic sagittal balance, including flat back deformity, stiff spine, abnormal spinopelvic tilt, and pelvic incidence(PI)-LL mismatch, which impairs stability and increases energy expenditure [25, 33]. As lumbar degenerative diseases and hip pathologies can reduce LL and SS, further causing increased PT to compensate, the preoperative plan of spinopelvic disturbance is imperative [34]. Despite the difference in surgical order, the postoperative spinopelvic parameters did not differ significantly between the two groups [11, 25, 28, 29]. Besides, previous studies have shown that significant decreases in PT and increases in SS were observed in patients with THA before LSF, while no significant changes were observed in patients with LSF before THA [28]. For patients with THA before LSF, spine surgeons should consider how to minimize deleterious orientation changes to the PT, or to improve current apparent impingement tendencies by driving PT orientation changes to values more appropriate to the previous implant cup orientation [11]. For patients with spinal deformity, it has been reported that a high PT is associated with the risk of dislocation [35]. The strategic planning of acetabular cup orientation for impingement-free stability necessitates primary consideration of PT [11]. In addition, the deviations of the PT from the typical balanced spinal profile should be adjusted by making 0.7 modifications to the cup anteversion target per degree of PT change, and the possible PT changes owing to compensatory adjustments to the previous LSF should also be considered [11].

As the mispositioning of the acetabular cup is closely related to the dislocation after THA, an optimum acetabular cup is particularly important, especially for patients with LSF or stiff spines. The criteria of “safe zone,” proposed by Dr.Lewinnek, is a radiographic inclination of 30–50° and radiographic anteversion of 5–25° in the anterior pelvic plane. However, it has been confirmed that no universal safe zone can prevent dislocation for all patients undergoing THA [36]. About 14.2% of hips within the safe zone cannot meet the criteria for the functional safe zone [37]. With the development of THA navigation and robotic systems, the precise planning of the cup orientation and placement has become possible. Besides, biomechanical and dynamic imaging analysis can be used to explain the functional interaction of the spine-pelvic-hip complex in different postures. A patient-specific safe zone algorithm has been reported to optimize the orientations of the acetabular cup and femoral stem on the basis of the standing and sitting PT [38].

This study has several limitations. Firstly, the absence of randomized prospective studies means an increased risk of selection and recall bias. Secondly, owing to a lack of sufficient data, some risk factors, such as radiologic parameters, fusion segments, fusion involving the sacrum, prosthesis types, and THA approach, were not included in the analysis. Lastly, although subgroup analysis has been conducted, high heterogeneity is often inevitable in meta-analysis.

## Conclusions

The rates of revision, PJI, dislocation, and aseptic loosening were comparable between patients undergoing THA after LSF and those undergoing LSF after THA. This suggests that there is no big difference between which LSF and THA perform first. Considering that patients undergoing both LSF and THA tend to have higher complication rates, a detailed preoperative plan for THA and LSF for sagittal alignment is necessary on the basis of the spine-pelvic-hip kinematics.

## Supplementary Information

Supplementary Material 1.

Supplementary Material 2.

Supplementary Material 3.

## Data Availability

All the data and material can be found in the article and the supplement materials.
