# Supplementary material for: Does the surgical sequence of lumbar spine fusion and total hip arthroplasty affect the dislocation and revision risk? A meta‑analysis and systematic review
Source: J Orthop Traumatol. 2026 Jun 12;27:45. doi: 10.1186/s10195-026-00943-5 (PMC13490027; doi:10.1186/s10195-026-00943-5)
Supplement: Supplementary file 1 [file 10195_2026_943_MOESM1_ESM.doc]

Supplement file 1 Quality evaluation of the eligible studies with MINORS

| **Study** | Clearly stated aim | Inclusion of consecutive patients | Prospective collection of data | Endpoints appropriate to study aims | Unbiased assessment of endpoints | Follow-up period appropriate | Loss to follow-up less than 5% | Prospective calculation of study size | Adequate control group | Contemporary groups | Baseline equivalence of groups | Adequate statistical analyses | Total |
| --- | --- | --- | --- | --- | --- | --- | --- | --- | --- | --- | --- | --- | --- |
| **Bala 2019** | 2 | 2 | 1 | 2 | 1 | 2 | 0 | 0 | 2 | 2 | 1 | 2 | 17 |
| **Grammatopoulos 2019** | 2 | 2 | 2 | 2 | 2 | 2 | 0 | 0 | 2 | 2 | 2 | 2 | 20 |
| **Parilla 2019** | 2 | 2 | 1 | 2 | 1 | 2 | 1 | 0 | 2 | 2 | 0 | 2 | 17 |
| **Yang 2020** | 2 | 2 | 1 | 2 | 0 | 2 | 0 | 0 | 2 | 2 | 1 | 2 | 16 |
| **Eneqvist 2024** | 2 | 2 | 2 | 2 | 1 | 2 | 0 | 0 | 2 | 2 | 1 | 2 | 18 |
| **Andah 2021** | 2 | 2 | 1 | 2 | 1 | 2 | 0 | 0 | 2 | 2 | 1 | 2 | 17 |
| **Welling 2023** | 2 | 2 | 1 | 2 | 1 | 2 | 0 | 0 | 2 | 2 | 1 | 2 | 17 |
| **Fan 2025** | 2 | 2 | 1 | 2 | 1 | 2 | 1 | 0 | 2 | 2 | 1 | 2 | 18 |
| **Mohamed 2023** | 2 | 2 | 1 | 2 | 0 | 2 | 0 | 0 | 2 | 2 | 1 | 2 | 16 |
| **Zhang 2023** | 2 | 2 | 1 | 2 | 1 | 2 | 0 | 0 | 2 | 2 | 0 | 2 | 16 |

无偏倚评估
