# Supplementary material for: Does the surgical sequence of lumbar spine fusion and total hip arthroplasty affect the dislocation and revision risk? A meta‑analysis and systematic review
Source: J Orthop Traumatol. 2026 Jun 12;27:45. doi: 10.1186/s10195-026-00943-5 (PMC13490027; doi:10.1186/s10195-026-00943-5)
Supplement: Supplementary file 2 [file 10195_2026_943_MOESM2_ESM.doc]

Supplement file3 Search stragety

| #1 | ("Total Hip Arthroplasty"[Title/Abstract] OR "Hip Replacement"[Title/Abstract] OR "Hip Arthroplasty"[Title/Abstract] OR "Hip Joint Replacement"[Title/Abstract] OR "Hip Joint Arthroplasty"[Title/Abstract] OR THA[Title/Abstract] OR THR [Title/Abstract]) |
| --- | --- |
| #2 | ("Lumbar Fusion"[Title/Abstract] OR "Spinal Fusion"[Title/Abstract] OR "Spine Fusion"[Title/Abstract] OR "Lumbar Vertebrae Fusion"[Title/Abstract] OR "Back Fusion"[Title/Abstract] OR "Lumbar Spondylodesis"[Title/Abstract] OR "Lumbar Interbody Fusion"[Title/Abstract] OR "Lumbar Arthrodesis"[Title/Abstract] OR "spine Arthrodesis"[Title/Abstract] OR "spinal Arthrodesis"[Title/Abstract] OR "Lumbar Interbody Arthrodesis"[Title/Abstract] OR "Lumbosacral Fusion"[Title/Abstract] OR "Lumbosacral Arthrodesis"[Title/Abstract] OR LSF[Title/Abstract] OR PLF[Title/Abstract] OR ALF[Title/Abstract] OR LIF[Title/Abstract]) |
| #3 | #1 AND #2 |
